# Irritable Bowel Syndrome and Incident Hypertension: Epidemiologic and Genetic Insights

**DOI:** 10.3390/biomedicines14092057

**Published:** 2026-09-13

**Authors:** Luyao Li, Changying Zhao, Feiyang Wang, Qian Zhao, Yan Zhao, Shouping Gong, Guoliang Li, Jing Li

**Affiliations:** 1 Department of Neurosurgery, The Second Affiliated Hospital of Xi’an Jiaotong University, 157 West 5th Road, Xi’an 710004, China; tina6918@stu.xjtu.edu.cn (L.L.); shpingg@126.com (S.G.); 2 Department of Cardiovascular Surgery, The First Affiliated Hospital of Xi’an Jiaotong University, 277 Yanta West Road, Xi’an 710061, China; zhaocy.susan@foxmail.com (C.Z.); wangfeiyang@xjtu.edu.cn (F.W.); 3 Department of Gastroenterology, The First Affiliated Hospital of Xi’an Jiaotong University, 277 Yanta West Road, Xi’an 710061, China; zhaoqian870419@163.com (Q.Z.); yanzhao211@163.com (Y.Z.); 4 Xi’an Medical University, No. 1 Xinwang Road, Xi’an 710021, China; 5 Department of Cardiovascular Medicine, The First Affiliated Hospital of Xi’an Jiaotong University, 277 Yanta West Road, Xi’an 710061, China; liguoliang_med@163.com; 6 Clinical Research Center, The First Affiliated Hospital of Xi’an Jiaotong University, 277 Yanta West Road, Xi’an 710061, China

**Keywords:** irritable bowel syndrome, hypertension, cohort study, *NCAM1*

## Abstract

**Background/Objectives**: Irritable bowel syndrome (IBS) may have health implications beyond the gastrointestinal tract, but its association with incident hypertension remains unclear. **Methods**: We analyzed 355,404 UK Biobank participants without recorded hypertension at baseline using multivariable Cox regression, propensity score matching, and sensitivity analyses addressing early events, baseline medication use and blood pressure, and competing mortality. Bidirectional Mendelian randomization (MR), Bayesian colocalization, and intestinal transcriptomic analyses examined genetic and tissue-level evidence. **Results**: During a median follow-up of 14.09 years, IBS was associated with incident hypertension (HR, 1.26; 95% CI, 1.22–1.30). The association persisted after matching (HR, 1.22; 95% CI, 1.16–1.28) and across sensitivity analyses. Forward MR provided suggestive support: the primary inverse-variance weighted estimate was positive but imprecise (OR, 1.18; 95% CI, 0.78–1.77), while weighted median and outlier-corrected analyses showed positive associations. Reverse MR showed no clear association. SuSiE-based colocalization supported shared signals for IBS with diastolic and systolic blood pressure at the *NCAM1* locus (PP.H4, 0.903 and 0.890, respectively). Intestinal *NCAM1* expression was lower in IBS, and correlated transcriptional programs involved epithelial remodeling, adhesion, and mucosal immune processes. **Conclusions**: IBS was associated with increased long-term hypertension risk, with suggestive genetic support and shared susceptibility signals at *NCAM1*. These findings support a potential biological link and prioritize *NCAM1* for mechanistic investigation.

## 1. Introduction

Irritable bowel syndrome (IBS) is a common disorder of gut–brain interaction affecting approximately 10% of the global population [1]. It is characterized by recurrent abdominal pain and altered bowel habits without an identifiable structural explanation [2]. Although primarily recognized by its gastrointestinal symptoms, IBS is frequently accompanied by extraintestinal manifestations [3,4], and low-grade systemic inflammation has been reported in some patient groups [5]. Large-scale genome-wide association studies (GWAS) have further reinforced this systemic perspective, identifying susceptibility loci—including *NCAM1*, *CADM2*, and *DOCK9*—that implicate neural and regulatory pathways beyond the gut [6]. These observations raise the possibility that IBS shares biological processes with conditions beyond the gastrointestinal tract.

Evidence linking IBS to blood pressure regulation is emerging. Genetic correlation analyses have demonstrated shared heritability between IBS and hypertension [7], and clinical studies have described masked hypertension and metabolic syndrome in people with IBS [8,9]. However, prevalent disease associations do not establish whether IBS precedes hypertension or whether shared risk factors account for the relationship. Prospective evidence is therefore needed, together with complementary genetic analyses to examine directionality and identify shared susceptibility loci.

Hypertension is a major contributor to cardiovascular disease and premature mortality [10]. Determining whether IBS is associated with its onset would help clarify the long-term health implications of IBS and establish whether further evaluation of IBS as a risk marker is warranted. Moreover, a combination of prospective epidemiology, genetic analysis, and intestinal transcriptomics can also help identify biological pathways that may connect gut dysfunction with vascular regulation [11].

We therefore examined the association between baseline IBS and incident hypertension in a prospective cohort, assessed genetic directionality using bidirectional Mendelian randomization, and investigated shared loci through colocalization. Intestinal mucosal transcriptomic analyses were then used to explore the biological context of the prioritized gene.

## 2. Materials and Methods

### 2.1. Study Population, Disease Ascertainment, and Follow-Up

**Figure 1:**
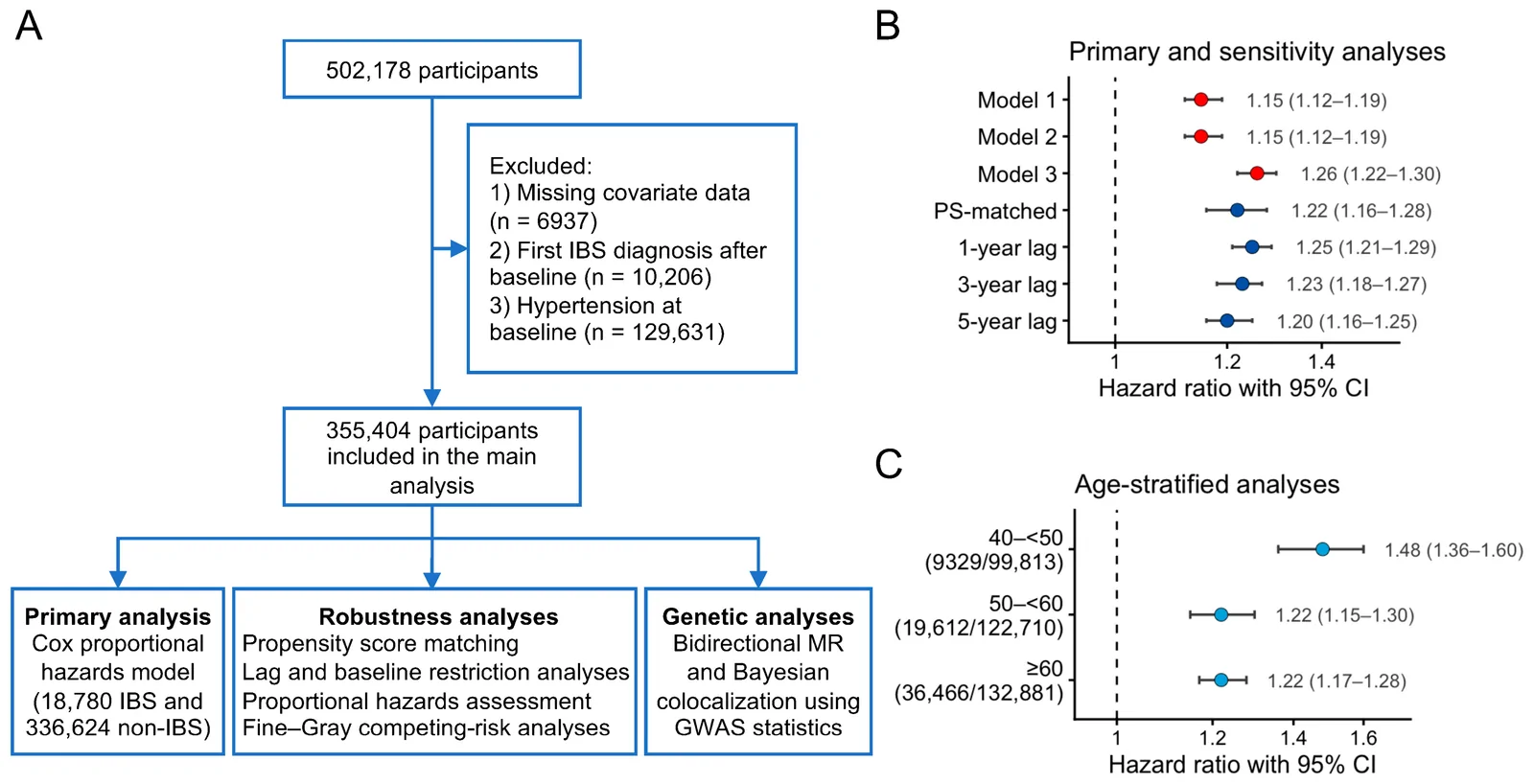
Study design and epidemiologic associations between irritable bowel syndrome and incident hypertension. (**A**) Overview of cohort derivation and the analytical framework, including the primary cohort, robustness, and genetic analyses. (**B**) Primary and sensitivity analyses of the association between baseline IBS and incident hypertension. Model 1 was adjusted for age and sex. Model 2 was additionally adjusted for educational attainment, Townsend deprivation index, and smoking status. Model 3 was further adjusted for body mass index, diabetes, hyperlipidemia, lipid-lowering medication use, and baseline systolic and diastolic blood pressure. Propensity score-matched estimates were derived from the 1:1 matched cohort, and lagged analyses excluded hypertension events occurring within the first 1, 3, and 5 years after baseline. Red, dark blue, and light blue points represent estimates from the primary models, sensitivity analyses, and age-stratified analyses, respectively; horizontal lines indicate 95% confidence intervals. (**C**) Age-stratified associations according to age at recruitment. Models were adjusted for continuous age within each stratum and the covariates included in Model 3. Numbers in parentheses indicate incident events/participants. Points indicate hazard ratios, and horizontal lines indicate 95% confidence intervals. CI, confidence interval; HR, hazard ratio; IBS, irritable bowel syndrome; PSM, propensity score matching.

We analyzed data from the UK Biobank, a prospective cohort of 502,178 individuals aged 40–69 years recruited across England, Scotland, and Wales between 2006 and 2010 [12]. We excluded 6937 participants with missing baseline smoking status, body mass index, Townsend deprivation index, or blood pressure data (1.38% of the initial cohort). After further excluding 10,206 participants whose first recorded IBS diagnosis occurred after baseline and 129,631 with prevalent hypertension, 355,404 participants remained for analysis (Figure 1A).

IBS and essential hypertension were ascertained using the UK Biobank first-occurrence date and source-of-report fields for ICD-10 categories K58 and I10, respectively. These derived fields integrate available information from self-reported diagnoses, primary care, hospital inpatient records, and death records. Baseline IBS was defined by a first recorded occurrence on or before the baseline assessment. Prevalent hypertension was defined similarly, and incident hypertension was defined by a first recorded occurrence after baseline. Definitions and coding of the exposure, outcome, and covariates are provided in Table S1.

Follow-up time was calculated from baseline assessment to first diagnosis of hypertension, death, loss to follow-up, or administrative censoring, whichever occurred first. Administrative censoring dates were 31 March 2023 for England, 31 August 2022 for Scotland, and 31 May 2022 for Wales. Median follow-up was estimated using the reverse Kaplan–Meier method.

### 2.2. Cox Regression and Sensitivity Analyses

Associations between baseline IBS and incident hypertension were estimated using Cox regression. Model 1 adjusted for age and sex. Model 2 additionally adjusted for educational attainment, Townsend deprivation index, and smoking status. Model 3 further adjusted for body mass index, diabetes, hyperlipidemia, lipid-lowering medication use, and baseline systolic and diastolic blood pressure. Age-stratified analyses were conducted for participants aged 40 to <50, 50 to <60, and ≥60 years, retaining continuous age within each stratum.

The proportional hazards assumption was assessed in Model 3 using scaled Schoenfeld residuals with a Kaplan–Meier transformation of time, including covariate-specific and global tests. To examine variation in the IBS association over follow-up, we split follow-up at 5 and 10 years and fitted separate Cox models for the intervals 0–5, >5–10, and >10 years using start–stop survival data and the Model 3 covariates.

The primary Cox analysis estimated cause-specific hazards, with participants censored at death. We additionally fitted a Fine–Gray model using the cmprsk package (version 2.2-12), treating death before hypertension as a competing event and adjusting for the Model 3 covariates. Results were expressed as subdistribution hazard ratios (sHRs) with 95% confidence intervals.

Time-lag sensitivity analyses excluded hypertension events occurring within the first 1, 3, and 5 years after baseline. To address possible misclassification of pre-existing hypertension, additional analyses among participants with available medication information excluded baseline antihypertensive medication users and then further restricted the sample to those with baseline systolic blood pressure < 140 mmHg and diastolic blood pressure < 90 mmHg.

### 2.3. Propensity Score-Matched Analysis

To further reduce confounding, propensity score matching was performed using the MatchIt package (version 4.7.2). Propensity scores were estimated using logistic regression with the covariates included in Model 3. Participants with IBS were matched to those without IBS using 1:1 nearest-neighbor matching without replacement, with a caliper width of 0.2 standard deviations of the estimated propensity scores. Covariate balance was assessed using absolute standardized mean differences, with values < 0.10 indicating adequate balance. The matched analysis used a Cox model stratified by matched pair and incorporating matching weights, allowing each pair to have its own baseline hazard function.

### 2.4. Genetic Data Sources and Instrument Selection

For the Mendelian randomization and colocalization analyses, we used summary statistics from large genome-wide association studies (GWAS) of IBS, hypertension, and blood pressure traits. The primary IBS GWAS and the GWAS for systolic blood pressure (SBP), diastolic blood pressure (DBP), pulse pressure (PP) [13], and hypertension [14] are listed in Supplementary Table S6. For IBS, genetic instruments were selected at genome-wide significance (*p* < 5 × 10^−8^). Variants with ambiguous high-frequency palindromic alleles that could not be reliably harmonized were excluded before LD clumping. Independent variants were identified using the European reference panel from the 1000 Genomes Project, with an LD threshold of r^2^ < 0.001 within a 10,000 kb window. Instrument strength was evaluated using the F statistic, calculated as the squared ratio of the SNP-exposure effect estimate to its standard error. Approximate variance explained was estimated from the SNP effect estimate, standard error, and variant-specific sample size. After harmonization, 7 independent IBS-associated SNPs were available as instruments for forward MR. The seven IBS instruments had F statistics ranging from 30.51 to 39.51 (mean, 33.44) and collectively explained approximately 0.051% of the observed-scale exposure variance. Detailed lists of instruments and their characteristics are provided in Supplementary Table S7. For hypertension, 117 independent instruments were identified before harmonization, with F statistics ranging from 29.84 to 213.45 (mean, 50.91) and collectively explaining approximately 1.46% of the observed-scale exposure variance. Of these, 114 were retained after harmonization for reverse MR.

### 2.5. Mendelian Randomization Analyses

We performed bidirectional two-sample Mendelian randomization to examine the association of genetically proxied IBS liability with hypertension (forward MR) and that of genetically proxied hypertension liability with IBS (reverse MR). For binary exposures, MR estimates were scaled and reported as odds ratios for the outcome per doubling in genetically predicted odds of the exposure by multiplying log-odds estimates and standard errors by log(2). The primary estimates were obtained using the inverse-variance weighted (IVW) method under a multiplicative random-effects model. Sensitivity analyses included the weighted median estimator and the weighted mode estimator, which provide robust estimates under different patterns of invalid instruments.

MR-Egger regression was used as an additional sensitivity analysis, and its intercept was examined for evidence of directional horizontal pleiotropy. Heterogeneity across SNP-specific estimates was assessed using Cochran’s Q statistic. MR-PRESSO was performed using 10,000 simulations to assess horizontal pleiotropy and identify outlier variants [15]. Outlier-corrected estimates were reported, and the distortion test was used to assess whether estimates differed before and after outlier removal. Analyses were conducted in R using TwoSampleMR (version 0.6.9) [16] and MRPRESSO (version 1.0).

### 2.6. Colocalization Analysis

To evaluate whether IBS and blood pressure traits share signals at specific loci, we performed region-based Bayesian colocalization. We focused on genomic regions harboring known IBS susceptibility genes (including *PHF2/FAM120A*, *DOCK9*, *NCAM1*, *WNK2*, and *CADM2*), as identified in previous GWAS and by mapping the IBS instruments (Supplementary Table S10). For each candidate gene, the analysis region extended from 1 Mb upstream of the gene start to 1 Mb downstream of the gene end.

We used the coloc.abf function from the coloc package (version 5.2.3) [17] to compute posterior probabilities for five mutually exclusive hypotheses: no association with either trait (H0), association with only one trait (H1/H2), association with both traits driven by distinct variants (H3), or association with both traits driven by a shared causal variant (H4). Default prior probabilities were used for the main analysis, and we considered PP(H4) ≥ 0.7 as evidence of colocalization.

Prior sensitivity was assessed by varying the prior probability that a variant was associated with both traits (p12) and examining the resulting support for shared versus distinct association signals. To allow for multiple causal signals within the *NCAM1* region, we performed SuSiE-based colocalization for IBS with DBP and SBP [18] using susieR (version 0.14.2). GWAS effect estimates were harmonized to the allele orientation of a signed LD matrix from the 1000 Genomes European reference panel. Variants that could not be aligned were excluded, and shared variants were ordered identically across the summary statistics and LD matrix. Trait-specific signals were identified using coloc::runsusie with default settings and compared using coloc::coloc.susie, with prior probabilities of p1 = p2 = 1 × 10^−4^ and p12 = 1 × 10^−5^. Posterior probabilities were evaluated for individual signal pairs.

### 2.7. Transcriptomic Analysis

To explore the tissue context of the colocalization-prioritized locus, we analyzed the public intestinal mucosal transcriptomic dataset GSE166869, which includes duodenal and jejunal mucosal biopsies from healthy controls and patients with IBS [19]. Matching expression data to sample annotations yielded 69 mucosal samples. Protein-coding genes were retained, with *NCAM1* explicitly preserved; for duplicated gene symbols, the entry with the highest mean RPKM across samples was retained. Gene expression values were analyzed as log_2_(RPKM + 1). Differential expression analysis between IBS and healthy mucosal samples was performed using limma (version 3.60.6) [20], with intestinal region included as a covariate, followed by empirical Bayes moderation with a mean–variance trend. *p* values were adjusted across genes using the Benjamini–Hochberg method, with an FDR-adjusted *p* value < 0.05 defining statistical significance. Pairwise comparisons among IBS subtypes and healthy controls used estimated marginal means, with Benjamini–Hochberg adjustment across pairwise comparisons within each intestinal region.

To characterize *NCAM1*-associated transcriptional programs, genes were ranked according to their Spearman correlation coefficients with *NCAM1* expression across intestinal mucosal samples. Gene set enrichment analysis was performed using ranked gene lists, and representative Hallmark gene sets were interpreted using the Molecular Signatures Database framework [21,22]. Gene Ontology biological process enrichment analysis of IBS-associated differentially expressed genes was performed using clusterProfiler (version 4.12.0) [23], and enriched terms were summarized as pathway similarity networks. Furthermore, to evaluate the mucosal immune and stromal context associated with *NCAM1*, cell-type abundance scores were estimated from bulk transcriptomic profiles using MCPcounter (version 1.2.0) [24]. Associations between *NCAM1* expression and estimated mucosal cell-type abundance were assessed using linear models. Pearson correlations among estimated cell-type abundance scores and Mantel associations with *NCAM1* expression were visualized.

All analyses were performed in R (version 4.4.0), and all statistical tests were two-sided.

## 3. Results

### 3.1. IBS Is Associated with Increased Risk of Incident Hypertension

The primary prospective analysis included 355,404 participants without recorded hypertension at baseline, comprising 18,780 participants with IBS and 336,624 without IBS (Table 1 and Figure 1A). During a median follow-up of 14.09 years, 65,407 incident hypertension events were documented, including 3849 among participants with IBS and 61,558 among those without IBS.

In Cox proportional hazards models adjusted for age and sex (Model 1), IBS was associated with a higher risk of incident hypertension (HR, 1.15; 95% CI, 1.12–1.19). Further adjustment for educational attainment, Townsend deprivation index, and smoking status (Model 2) yielded a similar estimate (HR, 1.15; 95% CI, 1.12–1.19). In the fully adjusted model (Model 3), additionally accounting for BMI, diabetes, hyperlipidemia, lipid-lowering medication use, and baseline SBP and DBP, IBS remained associated with incident hypertension (HR, 1.26; 95% CI, 1.22–1.30). As reported in Table 2, the association remained robust after restricting the analysis to participants with available medication data who reported no baseline use of antihypertensive medication (HR, 1.28; 95% CI, 1.24–1.33). In a more stringent sensitivity analysis additionally excluding participants with measured baseline systolic blood pressure ≥ 140 mmHg or diastolic blood pressure ≥ 90 mmHg, the association remained essentially unchanged (HR, 1.27; 95% CI, 1.20–1.35).

### 3.2. Age-Stratified Association Between IBS and Hypertension

The association between IBS and incident hypertension was observed across all three age groups (Figure 1C and Table S2). The point estimate was largest among participants aged 40 to <50 years (HR, 1.48; 95% CI, 1.36–1.60), whereas similar estimates were observed among those aged 50 to <60 years (HR, 1.22; 95% CI, 1.15–1.30) and those aged ≥60 years (HR, 1.22; 95% CI, 1.17–1.28).

### 3.3. Sensitivity Analyses and Variation over Follow-Up

The association remained robust across several complementary sensitivity analyses. After excluding hypertension events occurring within the first 1, 3, and 5 years after baseline, the fully adjusted HRs were 1.25 (95% CI, 1.21–1.29), 1.23 (95% CI, 1.18–1.27), and 1.20 (95% CI, 1.16–1.25), respectively (Table S3).

Results were also consistent after balancing measured baseline characteristics through propensity score matching. The matched cohort included 18,780 pairs, comprising 37,560 participants, and achieved satisfactory covariate balance, with all absolute standardized mean differences below 0.02 (Table S4). In the Cox model stratified by matched pair and incorporating the matching weights, IBS remained associated with incident hypertension (HR, 1.22; 95% CI, 1.16–1.28; *p* < 0.001).

Given the long follow-up period, we further assessed whether the association varied over time. Schoenfeld residual tests indicated departures from the proportional hazards assumption for IBS status (*p* = 4.6 × 10^−5^) and for the model globally (*p* < 2 × 10^−16^). Consistent with this finding, the interval-specific HR decreased from 1.40 (95% CI, 1.32–1.48) during the first 5 years to 1.27 (95% CI, 1.21–1.34) during >5–10 years and 1.12 (95% CI, 1.05–1.19) beyond 10 years (Table S5). The overall Cox HR was therefore interpreted as a summary association across the entire follow-up period rather than as a constant relative hazard.

Finally, accounting for death before hypertension as a competing event yielded a similar result. In the Fine–Gray analysis, IBS was associated with a higher incidence of hypertension (sHR, 1.27; 95% CI, 1.23–1.32; *p* < 0.001; Table S5).

### 3.4. Bidirectional Mendelian Randomization Analyses

To complement the cohort findings, we performed bidirectional two-sample Mendelian randomization (Figure S1).

After stringent LD clumping, seven independent IBS-associated variants were included in the forward MR analysis (Table S7). The primary IVW estimate was positive but imprecise (OR, 1.18; 95% CI, 0.78–1.77; *p* = 0.434). The weighted median estimate was positive (OR, 1.37; 95% CI, 1.12–1.67; *p* = 0.002). The weighted mode estimate was directionally consistent (OR, 1.34; 95% CI, 1.05–1.70; *p* = 0.056), whereas the MR-Egger estimate was imprecise (Table 3). Substantial heterogeneity was observed across instruments (IVW Cochran’s Q = 71.88; *p* = 1.68 × 10^−13^). The MR-Egger intercept test was not significant (*p* = 0.631), although this does not exclude horizontal pleiotropy (Table S8).

MR-PRESSO identified two outlier variants, rs1052486 and rs12549729. After their exclusion, the estimate based on the remaining five instruments was positive (OR, 1.24; 95% CI, 1.05–1.47; *p* = 0.013), although the distortion test was not significant (*p* = 0.4566; Table S9). In the reverse analysis, 114 hypertension-associated instruments yielded an IVW OR of 1.01 (95% CI, 0.99–1.04; *p* = 0.28). Overall, the forward MR analyses provided suggestive, although method-dependent, support for a positive association between IBS liability and hypertension.

### 3.5. Bayesian Colocalization Prioritizes NCAM1 as a Shared Locus Between IBS and Blood Pressure Traits

We next investigated whether IBS and blood pressure traits shared genetic association signals within candidate regions. IBS-associated instruments were annotated to their nearest genes and previously reported IBS susceptibility regions, identifying candidate loci including *FAM120A/PHF2*, *FAM120AOS*, *DOCK9*, *NCAM1*, *WNK2*, and *CADM2* (Table S10). These loci were then evaluated for colocalization with SBP, DBP, and PP.

**Figure 2:**
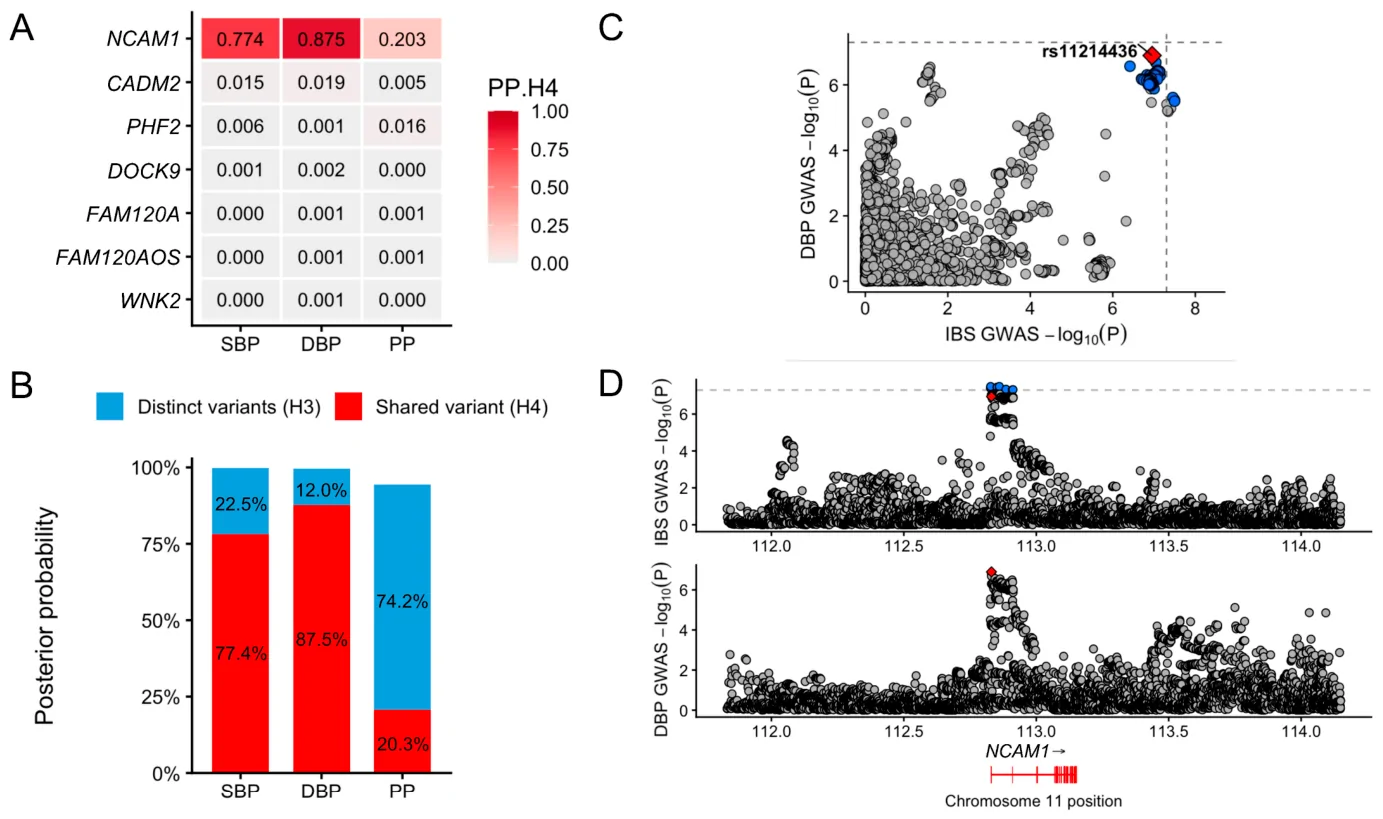
Bayesian colocalization analysis of IBS and blood pressure-related traits at the *NCAM1* locus. (**A**) Heat map showing posterior probabilities for a shared causal variant between IBS and blood pressure-related traits across candidate loci. (**B**) Posterior probability distribution at the *NCAM1* locus, comparing the probability of distinct causal variants and a shared causal variant between IBS and blood pressure-related traits. (**C**) SNP-level comparison of IBS and diastolic blood pressure GWAS association signals at the *NCAM1* locus. The leading regional association variant is highlighted. Dashed lines indicate the genome-wide significance threshold. (**D**) Regional association plots of IBS and diastolic blood pressure GWAS signals at the *NCAM1* locus. In panels (**C**) and (**D**), red diamonds denote the leading variants for the corresponding association signals, blue circles denote other variants within the highlighted shared association signal, and gray circles denote the remaining regional variants. DBP indicates diastolic blood pressure; GWAS, genome-wide association study; IBS, irritable bowel syndrome; *NCAM1*, neural cell adhesion molecule 1; PP, pulse pressure; PP.H4, posterior probability for a shared causal variant; SBP, systolic blood pressure; and SNP, single-nucleotide polymorphism.

Most candidate regions showed little evidence for a shared causal variant with blood pressure traits, with posterior probabilities generally favoring distinct nearby signals rather than colocalization (Table S11). *NCAM1* showed the strongest evidence of colocalization. At the *NCAM1* locus, posterior support for a shared causal variant was high for DBP (PP.H4 = 0.875) and SBP (PP.H4 = 0.774), whereas support for PP was weaker (PP.H4 = 0.203; Figure 2 and Figure S2). The same top blood pressure SNP was observed for SBP and DBP at this locus, further supporting *NCAM1* locus as the leading shared genetic signal between IBS and blood pressure-related traits in this analysis.

Prior-sensitivity analyses characterized how posterior support for H3 and H4 varied across the evaluated values of p12 (Figure S3). To account for multiple association signals within the *NCAM1* region, we additionally performed SuSiE-based colocalization using 5752 shared variants. The SBP analysis identified three signal pairs involving the IBS signal represented by rs7106434. Strong colocalization support was observed for the pair involving the SBP signal represented by rs11214436 (PP.H4 = 0.890; PP.H3 = 0.110), whereas the other two pairs predominantly supported distinct signals. The DBP analysis also supported colocalization between signals represented by rs7106434 and rs11214436 (PP.H4 = 0.903; Table S12). These findings support a shared association signal within the *NCAM1* region rather than implying that all blood pressure-associated signals in this region are shared with IBS.

### 3.6. NCAM1 Expression and Related Transcriptional Changes in IBS Intestinal Mucosa

**Figure 3:**
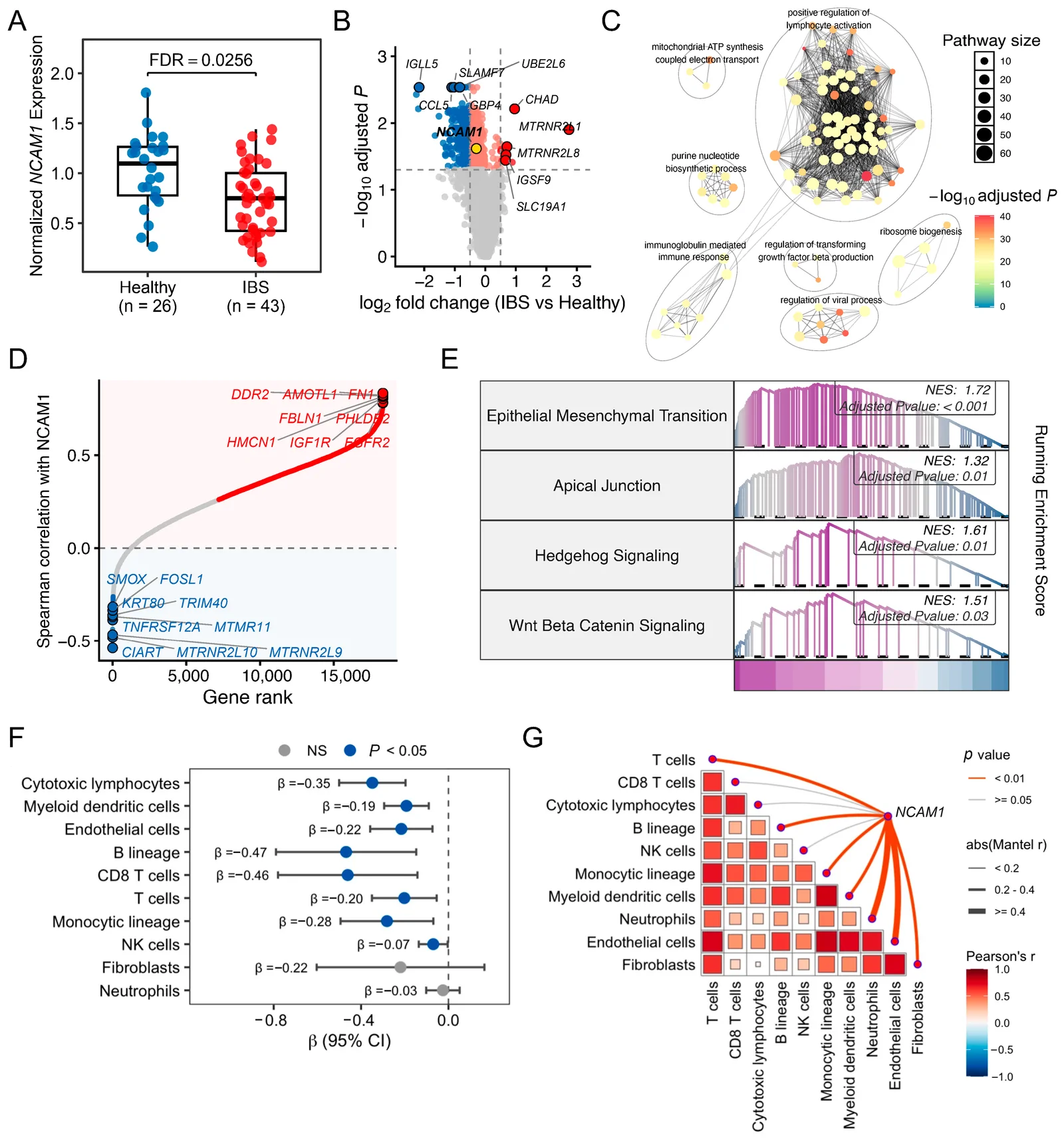
*NCAM1* expression and related transcriptional changes in IBS intestinal mucosa. (**A**) *NCAM1* expression in healthy and IBS intestinal mucosal samples. Box plots show the median and interquartile range, with individual samples overlaid. (**B**) Volcano plot of differentially expressed genes between IBS and healthy intestinal mucosa. *NCAM1* and the top 5 upregulated and top 5 downregulated genes are labeled. (**C**) Gene Ontology biological process enrichment network of IBS-associated differentially expressed genes. Node size indicates pathway size, node color indicates −log_10_(BH-adjusted *p*), and edges indicate similarity between enriched terms. (**D**) Genome-wide correlation landscape centered on *NCAM1*. Genes are ranked according to their correlation with *NCAM1* expression, and representative positively and negatively correlated genes are labeled. (**E**) Gene set enrichment curves for representative Hallmark pathways associated with *NCAM1*-correlated genes. (**F**) Associations between *NCAM1* expression and estimated mucosal cell-type abundance. Points indicate β coefficients, and horizontal lines indicate 95% CIs. (**G**) Pearson correlations among estimated mucosal cell-type abundance scores and Mantel associations with *NCAM1* expression. Link width represents the absolute Mantel correlation coefficient, and link color indicates the *p* value. CI indicates confidence interval; IBS, irritable bowel syndrome; and *NCAM1*, neural cell adhesion molecule 1.

To explore the disease-relevant tissue context of the prioritized *NCAM1* locus, we analyzed intestinal mucosal transcriptomic data from healthy controls and patients with IBS (GSE166869). In the pooled analysis adjusting for intestinal region, *NCAM1* expression was lower in IBS than in healthy mucosal samples (adjusted difference on the log_2_[RPKM + 1] scale, −0.291; FDR-adjusted *p* = 0.0256; Figure 3A). Expression patterns across IBS subtypes and healthy controls within each intestinal region are shown in Figure S4. Thus, *NCAM1* was not only a genetic signal but also showed altered expression in an IBS-relevant intestinal tissue context.

We next examined whether this expression change occurred within a broader mucosal transcriptional program. Differential expression analysis further showed broad IBS-associated transcriptional changes, with *NCAM1* highlighted within the overall expression landscape (Figure 3B). Gene Ontology enrichment of IBS-associated differentially expressed genes summarized the surrounding mucosal biology, identifying interconnected modules involving immune and humoral responses, ribosome biogenesis, and regulatory processes including TGF-β-related signaling (Figure 3C). These results provided the global IBS mucosal background against which *NCAM1*-related programs were interpreted.

To move from global IBS-associated changes to *NCAM1*-centered biology, genes were ranked according to their correlation with *NCAM1* expression. Positively correlated genes included extracellular matrix and adhesion-related genes such as *DDR2*, *FBLN1*, *AMOTL1*, and *FN1*, whereas negatively correlated genes included stress- and epithelial-related transcripts (Figure 3D). Consistently, Hallmark enrichment analysis of the *NCAM1*-ranked gene list highlighted epithelial–mesenchymal transition, apical junction, Hedgehog signaling, and WNT/β-catenin signaling programs (Figure 3E). Estimated cell-type abundance analyses further linked *NCAM1* expression with selected immune and stromal features in the mucosal microenvironment (Figure 3F,G). Taken together, these analyses suggest that *NCAM1* in IBS mucosa is embedded in a coordinated transcriptional context involving epithelial remodeling, cell adhesion, and mucosal immune–stromal organization.

## 4. Discussion

In this prospective cohort, IBS was associated with incident hypertension after adjustment for established risk factors and across multiple sensitivity analyses. The association attenuated over follow-up but remained evident beyond 10 years. MR provided suggestive genetic support, with positive weighted median and outlier-corrected estimates alongside an imprecise primary IVW estimate. Colocalization identified a shared signal for IBS and blood pressure traits at *NCAM1*, while intestinal transcriptomic analyses linked *NCAM1* expression to epithelial remodeling, adhesion, and mucosal immune processes. These complementary findings support further investigation of the biological connection between IBS and hypertension.

Our findings extend the clinical relevance of IBS beyond gastrointestinal symptoms. The association persisted after adjustment for baseline blood pressure and cardiometabolic risk factors, including in participants without antihypertensive medication use and with baseline blood pressure < 140/90 mmHg. IBS may therefore help identify a population with increased long-term hypertension risk. Whether it adds useful predictive information beyond established risk factors requires independent evaluation. Changes to hypertension screening or management would require validation in independent populations.

The relative association was strongest among participants younger than 50 years at recruitment. A lower background risk of hypertension may make the relative contribution of IBS-related factors more apparent at younger ages, whereas vascular aging and accumulated metabolic risk may increasingly dominate at older ages. The declining association over follow-up may reflect changes in IBS activity, treatment, lifestyle, or healthcare contact, although these explanations could not be tested here. The persistence of an association after excluding early events makes an explanation based solely on imminent hypertension less likely.

Several pathways may connect IBS with blood pressure regulation. Altered autonomic responses reported in IBS suggest that sympathetic and parasympathetic imbalance could influence both visceral function and vascular tone [25]. Mucosal mast-cell activation near enteric nerves provides evidence of neuroimmune signaling in IBS [26], offering a possible link between chronic low-grade inflammation and altered gut–brain responses. Gut microbial changes may also influence systemic vascular regulation: transfer of microbiota from hypertensive patients increased blood pressure in germ-free mice [27]. Experimental evidence that immune activation contributes to hypertension, vascular oxidative stress, and endothelial dysfunction provides a further potential connection [28]. These studies support plausible pathways, but their contribution to the association observed here remains to be established.

The MR results offer suggestive support for an association between IBS liability and hypertension. The IVW, weighted median, and outlier-corrected estimates were directionally aligned, although the primary IVW confidence interval included the null. With only seven independent instruments explaining a small proportion of variation in IBS status, substantial heterogeneity reduced the precision of the primary estimate. The positive weighted median and MR-PRESSO results are therefore informative, but sensitivity to the analytical method and possible residual pleiotropy preclude a firm causal conclusion. Reverse MR showed no clear association, although an effect in that direction cannot be excluded. Colocalization provides complementary evidence of shared genetic susceptibility, and does not itself establish the direction of causation.

The colocalization analysis prioritized *NCAM1* as the leading shared locus between IBS and blood pressure-related traits. This finding is notable because *NCAM1* was among the susceptibility loci identified in the large IBS GWAS, which highlighted shared genetic pathways between IBS and mood- and anxiety-related traits and supported the concept of IBS as a disorder involving gut–brain interaction [6]. *NCAM1* encodes neural cell adhesion molecule 1, a member of the immunoglobulin superfamily involved in cell adhesion, but its biological relevance is not limited to the nervous system [29]. In epithelial models, NCAM expression has been shown to promote focal adhesion assembly, cell spreading, delamination, and migration during loss of E-cadherin, linking NCAM biology to adhesion remodeling and epithelial–mesenchymal transition-like behavior [30]. NCAM1 also has immune relevance through its protein product CD56; recent experimental work showed that CD56/NCAM is required for human NK-cell migration on ICAM-1 by regulating actin polarity and integrin-mediated adhesion turnover [31]. These adhesion-related and immune-cell functions are consistent with our intestinal mucosal transcriptomic findings, in which *NCAM1*-correlated genes were linked to epithelial remodeling, cell adhesion, and mucosal immune features.

The intestinal mucosal transcriptomic analysis helped bridge the genetic signal to a disease-relevant tissue setting. *NCAM1* was modestly downregulated in IBS mucosa and was embedded within transcriptional programs involving epithelial remodeling, adhesion-related genes, WNT/Hedgehog signaling, and immune–stromal features. These findings provide a coherent mucosal context in which the *NCAM1* locus can be interpreted within IBS biology. This interpretation is consistent with prior small intestinal transcriptomic work in IBS, which linked mucosal gene-expression changes to TRPV channel expression, ion-channel transport, stimuli-sensing pathways, and symptom-related intestinal chemosensitivity [19]. Together, these observations raise the possibility that reduced intestinal *NCAM1* expression accompanies altered epithelial adhesion and barrier remodeling and may influence mucosal immune-cell trafficking and gut–vascular signaling; however, these mechanisms require functional validation.

Several limitations should be acknowledged. First, hypertension ascertainment relied on routinely collected records, which may have missed undiagnosed cases and may have been influenced by greater healthcare contact among participants with IBS. Nevertheless, the consistent findings after restricting the analyses according to baseline antihypertensive use and measured blood pressure reduce concern about misclassification of pre-existing hypertension. Second, antihypertensive treatment initiated during follow-up and lifestyle changes after IBS diagnosis were not modeled, and residual confounding cannot be entirely excluded. Complete-case analysis may also introduce selection bias, although only 1.38% of participants were excluded because of missing covariates. Third, the healthy-volunteer profile of UK Biobank and the predominantly European ancestry of the study populations may limit generalizability [32]. The genetic findings nevertheless warrant cautious interpretation, as the MR analyses were based on a limited number of IBS instruments and showed heterogeneity, leaving open the possibility of residual pleiotropy. Although the colocalization and intestinal transcriptomic results converged on *NCAM1*, these analyses cannot establish the causal gene or underlying mechanism. Moreover, the bulk-tissue data did not permit cell-specific inference or adjustment for within-participant correlations among biopsies. Independent replication and functional validation will help define the biological and clinical relevance of these findings.

## 5. Conclusions

IBS was associated with increased incident hypertension risk in a large prospective cohort, with suggestive support from MR and shared genetic signals at the *NCAM1* locus. Intestinal transcriptomic findings further implicated epithelial remodeling, cell adhesion, and mucosal immune processes. Together, these findings support a potential biological link between IBS and hypertension and prioritize *NCAM1* for mechanistic investigation. Independent validation will help establish their clinical relevance.

## Figures and Tables

**Table 1:** Baseline characteristics of participants free of hypertension at baseline.

Characteristic	IBS (*n* = 18,780)	Non-IBS (*n* = 336,624)	SMD ^1^
Age, y	55.20 (7.96)	55.40 (8.17)	0.024
Sex			0.004
Male	8611 (45.9%)	153,606 (45.6%)	
Female	10,169 (54.1%)	183,018 (54.4%)	
Education level			0.054
College or university degree	6043 (32.2%)	116,950 (34.7%)	
Others	12,737 (67.8%)	219,674 (65.3%)	
Socioeconomic level ^2^			0.014
Least deprived	4934 (26.3%)	86,483 (25.7%)	
Moderately deprived	9401 (50.0%)	169,400 (50.3%)	
Most deprived	4445 (23.7%)	80,741 (24.0%)	
Smoking status			0.019
Current	1946 (10.4%)	36,851 (10.9%)	
Previous	6153 (32.8%)	109,582 (32.6%)	
Never	10,681 (56.9%)	190,191 (56.5%)	
BMI, kg/m^2^	26.50 (4.65)	26.70 (4.39)	0.045
Diabetes	99 (0.5%)	1946 (0.6%)	0.007
Hyperlipidemia	2347 (12.5%)	37,014 (11.0%)	0.046
Lipid-lowering medication use	1349 (7.2%)	26,075 (7.7%)	0.021
SBP, mmHg	133.44 (17.95)	136.41 (18.62)	0.163
DBP, mmHg	79.42 (10.00)	80.85 (10.27)	0.141

^1^ Standardized mean differences (SMDs) were used to quantify the magnitude of baseline differences between participants with and without irritable bowel syndrome, with an SMD < 0.1 generally considered a negligible difference. ^2^ Socioeconomic level was assessed using the Townsend deprivation index (TDI), with higher scores indicating greater area-level deprivation. TDI was categorized into three groups: least deprived (quartile 1), moderately deprived (quartile 2–3), and most deprived (quartile 4). SBP, systolic blood pressure. DBP, diastolic blood pressure. Data are presented as mean (SD) or n (%).

**Table 2:** Associations between baseline IBS and incident hypertension using multivariable Cox proportional hazards models.

Analysis	Participants Included ^1^, *n* (Incident Cases)	Model 1 ^2^		Model 2 ^3^		Model 3 ^4^	
		HR (95% CI)	*p* Value	HR (95% CI)	*p* Value	HR (95% CI)	*p* Value
Primary analysis	355,404 (65,407)	1.15 (1.12–1.19)	4.29 × 10^−18^	1.15 (1.12–1.19)	1.32 × 10^−17^	1.26 (1.22–1.30)	2.33 × 10^−44^
No baseline antihypertensive use	344,286 (59,283)	1.17 (1.13–1.21)	2.87 × 10^−19^	1.16 (1.13–1.20)	1.42 × 10^−18^	1.28 (1.24–1.33)	2.24 × 10^−47^
No baseline antihypertensive use and BP < 140/90 mmHg	198,347 (17,753)	1.29 (1.22–1.37)	4.50 × 10^−19^	1.28 (1.21–1.35)	8.65 × 10^−18^	1.27 (1.20–1.35)	4.28 × 10^−17^

^1^ Participants included are individuals free of hypertension at baseline and who had complete data for the covariates included in the analysis. ^2^ Model 1 adjusted for age and sex. ^3^ Model 2 further adjusted for educational attainment (college or university degree, yes/no), Townsend deprivation index (continuous), and smoking status (never, previous, current). ^4^ Model 3 additionally adjusted for BMI, diabetes, hyperlipidemia, use of lipid-lowering medications, and baseline blood pressure (SBP and DBP). IBS, irritable bowel syndrome; HR, hazard ratio; CI, confidence interval.

**Table 3:** Bidirectional Mendelian randomization results.

Exposure	Outcome	Instruments, *n*	IVW		Weighted Median		Weighted Mode		MR-Egger	
			OR (95% CI)	*p* Value	OR (95% CI)	*p* Value	OR (95% CI)	*p* Value	OR (95% CI)	*p* Value
IBS	Hypertension	7	1.18 (0.78–1.77)	0.434	1.37 (1.12–1.67)	0.002	1.34 (1.05–1.70)	0.056	0.29 (0.001–66.96)	0.671
Hypertension	IBS	114	1.01 (0.99–1.04)	0.28	1.00 (0.98–1.04)	0.73	0.99 (0.92–1.05)	0.67	1.03 (0.95–1.12)	0.47

IBS, irritable bowel syndrome; MR, Mendelian randomization; SNP, single-nucleotide polymorphism; IVW, inverse-variance weighted. OR: MR estimates are presented as odds ratios for the outcome per doubling in genetically predicted odds of the exposure.

## Data Availability

The UK Biobank dataset is available to qualified researchers via application to the UK Biobank (http://ukbiobank.ac.uk/register-apply/ (accessed on 9 September 2026)). This research was conducted under license from the UK Biobank (application number 152047). Summary statistics for the IBS GWAS and blood pressure GWAS used in this study are available from the cited publications and are listed in Supplementary Table S6. The intestinal mucosal transcriptomic dataset is available from the Gene Expression Omnibus under accession number GSE166869 (https://www.ncbi.nlm.nih.gov/geo/query/acc.cgi?acc=GSE166869 (accessed on 9 September 2026)). The analysis code will be made available upon reasonable request.

## Supplementary Materials

The following supporting information can be downloaded at: https://www.mdpi.com/article/10.3390/biomedicines14092057/s1, Table S1. Definitions and coding of IBS, hypertension and covariates; Table S2. Age-stratified association between IBS and incident hypertension; Table S3. Time-lag sensitivity analysis of the association between baseline IBS and incident hypertension; Table S4. Baseline characteristics of the study participants after propensity score matching; Table S5. Additional analyses evaluating the robustness and temporal pattern of the association between IBS and incident hypertension; Table S6. Data sources for Mendelian randomization and colocalization analyses; Table S7. Genetic instruments used for Mendelian randomization of IBS on hypertension; Table S8. Heterogeneity and pleiotropy statistics for bi-directional Mendelian randomization; Table S9. MR-PRESSO sensitivity analysis for the association between IBS and hypertension; Table S10. Nearest-gene annotation for IBS-associated SNPs; Table S11. Bayesian colocalization results for IBS and blood pressure-related traits across candidate loci; Table S12. SuSiE-based multi-signal colocalization analysis at the *NCAM1* locus; Figure S1. Mendelian randomization analyses and sensitivity analyses; Figure S2. Colocalization plot of *NCAM1* and systolic blood pressure; Figure S3. Prior sensitivity of colocalization at the *NCAM1* locus; Figure S4. *NCAM1* expression across IBS subtypes in duodenal and jejunal mucosal samples.
