# Supplementary material for: Irritable Bowel Syndrome and Incident Hypertension: Epidemiologic and Genetic Insights
Source: Biomedicines. 2026 Sep 13;14(9):2057. doi: 10.3390/biomedicines14092057 (PMC13604292; doi:10.3390/biomedicines14092057)
Supplement: Supplementary file 1 [file biomedicines-14-02057-s001.zip › biomedicines-4484065-supplementary.pdf]

Supplementary Materials

Table S1. Definitions and coding of IBS, hypertension and covariates.
Table S2. Age-stratified association between IBS and incident hypertension.
Table S3. Time-lag sensitivity analysis of the association between baseline IBS and incident hypertension.
Table S4. Baseline characteristics of the study participants after propensity score matching.
Table S5. Additional analyses evaluating the robustness and temporal pattern of the association between IBS and incident hypertension.
Table S6. Data sources for Mendelian randomization and colocalization analyses.
Table S7. Genetic instruments used for Mendelian randomization of IBS on hypertension.
Table S8. Heterogeneity and pleiotropy statistics for bi-directional Mendelian randomization.
Table S9. MR-PRESSO sensitivity analysis for the association between IBS and hypertension.
Table S10. Nearest-gene annotation for IBS-associated SNPs.
Table S11. Bayesian colocalization results for IBS and blood pressure-related traits across candidate loci.
Table S12. SuSiE-based multi-signal colocalization analysis at the *NCAM1* locus.

Figure S1. Mendelian randomization analyses and sensitivity analyses.
Figure S2. Colocalization plot of *NCAM1* and systolic blood pressure.
Figure S3. Prior sensitivity of colocalization at the *NCAM1* locus.
Figure S4. *NCAM1* expression across IBS subtypes in duodenal and jejunal mucosal samples.

Table S1. Definitions and coding of IBS, hypertension and covariates.

Variable	Definition	ICD-10	Data field ID in UK Biobank
**IBS**	IBS was defined using the date of first occurrence of ICD-10 K58 (Field 131638) and the corresponding source-of-report field (Field 131639), which identifies the data source(s) contributing to the first-occurrence record.	K58	131638, 131639
**Hypertension**	Incident hypertension was defined as the first occurrence of ICD-10 I10 after baseline. Field 131286 provided the date of first report, and Field 131287 provided the corresponding source of report.	I10	131286, 131287
**Age**	Age at recruitment (years), derived from date of birth and date of attending the initial assessment centre.		21022
**Sex**	Biological sex at baseline (male/female).		31
**Education Level**	Highest educational qualification at baseline		6138
**Socioeconomic Level**	Townsend deprivation index at recruitment (higher values indicate greater area-level deprivation).		189
**Smoking status**	Smoking status at baseline, derived variable summarising current/past smoking (never / previous / current) from the touchscreen questionnaire.		20116
**BMI, kg/m^2**	Body mass index at baseline, kg/m², derived from measured height and weight		21001
**Diabetes**		E10, E11, E13, E14	130706, 130708, 130712, 130714
**Hyperlipidemia**		E78	130814
**Lipid-lowering medication use**	Baseline lipid-lowering medication use, identified from statin-related medication codes in field 20003 (e.g., simvastatin, atorvastatin, pravastatin, rosuvastatin) or self-reported use of cholesterol-lowering medication in fields 6153 and 6177.		20003, 6153, 6177
**SBP, mmHg**	Systolic blood pressure at baseline (automated or manual if automated missing)		4080, 93
**DBP, mmHg**	Diastolic blood pressure at baseline (automated or manual if automated missing)		4079, 94
**Anti-hypertensive medication use**	Baseline antihypertensive medication use, identified from recorded medications (field 20003) or self-reported use of blood pressure-lowering medication (fields 6153 and 6177).		20003, 6153, 6177
**Death**	Date of death obtained through linkage to national death registries; used as the censoring date in the Cox analyses and to define death before hypertension as a competing event in the Fine–Gray analysis.		40000

Table S2. Age-stratified association between IBS and incident hypertension.

Age band	N(events/total)	Event rate	HR (95% CI)	*P* value
40–49	9,329/99,813	9.35%	1.48 (1.36–1.60)	1.07×10^{-20}
50–59	19,612/122,710	16.0%	1.22 (1.15–1.30)	9.43×10^{-12}
≥60	36,466/132,881	27.4%	1.22 (1.17–1.28)	1.18×10^{-18}

Table S3. Time-lag sensitivity analysis of the association between baseline IBS and incident hypertension.

Partici-pants included[a]	Incident cases	Model 3[b]		Lag years
		HR (95% CI)	*P*	
355,404	65,407	1.26 (1.22–1.30)	2.72×10^{-44}	0
351,194	61,664	1.25 (1.21–1.29)	1.15×10^{-37}	1
342,343	54,551	1.23 (1.18–1.27)	8.07×10^{-29}	3
331,517	45,785	1.20 (1.16–1.25)	5.49×10^{-20}	5

Lag years indicates the minimum number of years after baseline IBS diagnosis required before incident hypertension was counted (0 = primary analysis; 1, 3, and 5 years represent lagged sensitivity analyses). a. Participants included refers to the number of individuals with hypertension-free status at baseline in each lag cohort. b. Model 3 additionally adjusted for BMI, diabetes, hyperlipidemia, use of lipid-lowering medications, and baseline blood pressure (SBP and DBP).

Table S4. Baseline characteristics of the study participants after propensity score matching.

**Characteristic**	**IBS (*n*=18,780)**	**Non-IBS (*n*=18,780)**	**SMD[a]**
Age, y	55.20 (7.96)	55.20 (8.17)	0.006
Sex			0.007
Male	8,611 (45.9%)	8,676 (46.2%)	
Female	10,169 (54.1%)	10,104 (53.8%)	
Education level			<0.001
College or university degree	6,043 (32.2%)	6,045 (32.2%)	
Others	12,737 (67.8%)	12,735 (67.8%)	
Socioeconomic level[b]			0.008
Least deprived	4,934 (26.3%)	4,875 (26.0%)	
Moderate deprived	9,401 (50.1%)	9,417 (50.1%)	
Most deprived	4,445 (23.7%)	4,488 (23.9%)	
Smoking status			0.018
Current	1,946 (10.4%)	1,852 (9.9%)	
Previous	6,153 (32.8%)	6,255 (33.3%)	
Never	10,681 (56.9%)	10,673 (56.8%)	
BMI, kg/m^2	26.50 (4.65)	26.53 (4.33)	0.008
Diabetes	99 (0.5%)	105 (0.6%)	0.006
Hyperlipidemia	2,347 (12.5%)	2,378 (12.7%)	0.005
Lipid-lowering medication use	1,349 (7.2%)	1,347 (7.2%)	<0.001
SBP, mmHg	133.44 (17.95)	133.54 (17.98)	0.006
DBP, mmHg	79.42 (10.00)	79.40 (10.01)	0.002

Data are presented as mean ± standard deviation for continuous variables and number (percentage) for categorical variables. a. SMD denotes the absolute standardized mean difference between IBS and non-IBS groups after propensity score matching; values <0.10 were considered indicative of good balance; in the matched cohort, all the absolute SMDs were <0.02. b. Socioeconomic level was assessed using the Townsend deprivation index (TDI), with higher scores indicating greater area-level deprivation. TDI was categorized into three groups: least deprived (quartile 1), moderately deprived (quartile 2-3), and most deprived (quartile 4). SBP, systolic blood pressure. DBP, diastolic blood pressure.

Table S5. Additional analyses evaluating the robustness and temporal pattern of the association between IBS and incident hypertension.

Analysis	Estimate (95% CI)	*P* value
Fine–Gray competing risk model (death as competing event)	sHR 1.27 (1.23–1.32)	<0.001
0–5 year follow-up	HR 1.40 (1.32–1.48)	<0.001
>5–10 year follow-up	HR 1.27 (1.21–1.34)	<0.001
>10 year follow-up	HR 1.12 (1.05–1.19)	<0.001

Interval-specific estimates are hazard ratios from Cox models fitted to start–stop survival data for the indicated follow-up periods. The Fine–Gray estimate is a subdistribution hazard ratio, with death before incident hypertension treated as a competing event. All models were adjusted for the covariates included in Model 3. CI, confidence interval; HR, hazard ratio; sHR, subdistribution hazard ratio.

Table S6. Data sources for Mendelian randomization and colocalization analyses.

Trait	URL	Sample size	Reference	Publication year
**Irritable bowel syndrome**	https://www.ebi.ac.uk/gwas/studies/GCST90016564	486,601	PMID: 34741163	2021
**Hypertension**	https://www.ebi.ac.uk/gwas/studies/GCST90436066	408,089	PMID: 30104761	2018
**Systolic blood pressure**	https://www.ebi.ac.uk/gwas/studies/GCST90310294	1,028,980	PMID: 38689001	2024
**Diastolic blood pressure**	https://www.ebi.ac.uk/gwas/studies/GCST90310295	1,028,980	PMID: 38689001	2024
**Pulse pressure**	https://www.ebi.ac.uk/gwas/studies/GCST90310296	1,028,980	PMID: 38689001	2024

Table S7. Genetic instruments used for Mendelian randomization of IBS on hypertension.

SNP	Effect allele	Other allele	EAF	β	SE	*P* value	F statistic	R^2 (%)
rs1248825	A	C	0.3253	0.0444	0.0073	1.204×10^{-9}	36.99	0.0076
rs1052486	A	G	0.525	−0.044	0.007	4.178×10^{-10}	39.51	0.0084
rs12549729	T	G	0.3898	0.0475	0.0086	2.881×10^{-8}	30.51	0.0086
rs10156602	A	G	0.633	0.0417	0.0071	4.355×10^{-9}	34.49	0.0071
rs7106434	T	C	0.4095	0.0384	0.0069	3.189×10^{-8}	30.97	0.0064
rs2813573	T	C	0.4846	−0.0378	0.0068	3.308×10^{-8}	30.90	0.0064
rs9513519	A	G	0.6168	0.0388	0.007	3.087×10^{-8}	30.72	0.0063

Abbreviations: EAF, effect allele frequency; SE, standard error. F statistics were calculated as $(\beta/SE)^2$ to evaluate the strength of genetic instruments. All instrumental variables were selected after linkage disequilibrium clumping.

Table S8. Heterogeneity and pleiotropy statistics for bi-directional Mendelian randomization.

Direction	Q_IVW	df	*P*_het_IVW[a]	Q_Egger	df	*P*_het_Egger[b]	Egger intercept	SE	*P*_pleio[c]
IBS→Hypertension	71.88	6	1.68×10^{-13}	68.32	5	2.29×10^{-13}	0.0594	0.1163	0.631
Hypertension→IBS	179.21	113	7.31×10^{-5}	178.94	112	6.02×10^{-5}	−0.0014	0.0033	0.677

a. *P* value for heterogeneity from Cochran's Q statistic in the IVW model. b. *P* value for heterogeneity from Cochran's Q statistic in the MR-Egger regression. c. *P* value for directional horizontal pleiotropy from the MR-Egger intercept test. IBS, irritable bowel syndrome; IVW, inverse-variance weighted.

Table S9. MR-PRESSO sensitivity analysis for the association between IBS and hypertension.

Direction	Analysis	SNPs included	OR (95% CI)	*P* value
IBS→Hypertension	Raw MR-PRESSO analysis	7	1.18 (0.78–1.77)	0.464
IBS→Hypertension	Outlier-corrected MR-PRESSO analysis	5	1.24 (1.05–1.47)	0.013

MR-PRESSO was performed using 10,000 simulations. Two outlier variants (rs1052486 and rs12549729) were identified in the IBS-to-hypertension analysis. The distortion test showed no significant difference after outlier removal (P = 0.4566).

Table S10. Nearest-gene annotation for IBS-associated SNPs.

SNP	Chromosome	Position (bp)	Nearest gene	Ensembl gene ID	Distance to nearest gene (bp)
rs564	9	93566021	*FAM120A*	ENSG00000048828	0
rs1055710	9	93452646	*FAM120A*	ENSG00000048828	0
rs10512225	9	93464818	*FAM120A*	ENSG00000048828	0
rs10761226	9	93461754	*FAM120A*	ENSG00000048828	0
rs10761227	9	93464229	*FAM120A*	ENSG00000048828	0
rs10761228	9	93473303	*FAM120A*	ENSG00000048828	0
rs10761229	9	93475094	*FAM120A*	ENSG00000048828	0
rs10761230	9	93484276	*FAM120A*	ENSG00000048828	0
rs10761238	9	93588935	*PHF2*	ENSG00000197724	0
rs10821128	9	93447661	*FAM120AOS*	ENSG00000188938	0
rs10821129	9	93460335	*FAM120A*	ENSG00000048828	0
rs10821130	9	93461290	*FAM120A*	ENSG00000048828	0
rs10821134	9	93475936	*FAM120A*	ENSG00000048828	0
rs10821136	9	93476449	*FAM120A*	ENSG00000048828	0
rs10821137	9	93478751	*FAM120A*	ENSG00000048828	0
rs10821138	9	93485479	*FAM120A*	ENSG00000048828	0
rs10821139	9	93486851	*FAM120A*	ENSG00000048828	0
rs10821159	9	93552115	*FAM120A*	ENSG00000048828	0
rs10821160	9	93563845	*FAM120A*	ENSG00000048828	0
rs10992746	9	93468889	*FAM120A*	ENSG00000048828	0
rs10992748	9	93470460	*FAM120A*	ENSG00000048828	0
rs10992749	9	93470592	*FAM120A*	ENSG00000048828	0
rs10992751	9	93476808	*FAM120A*	ENSG00000048828	0
rs10992754	9	93483405	*FAM120A*	ENSG00000048828	0
rs10992783	9	93548974	*FAM120A*	ENSG00000048828	0
rs10992790	9	93579075	*PHF2*	ENSG00000197724	0
rs11790060	9	93440650	*FAM120AOS*	ENSG00000188938	0
rs11791374	9	93483005	*FAM120A*	ENSG00000048828	0
rs7869969	9	93455165	*FAM120A*	ENSG00000048828	0
rs10114002	9	93595748	*PHF2*	ENSG00000197724	0
rs10116422	9	93595457	*PHF2*	ENSG00000197724	0
rs10117691	9	93595643	*PHF2*	ENSG00000197724	0
rs4743922	9	93472410	*FAM120A*	ENSG00000048828	0
rs4744240	9	93455912	*FAM120A*	ENSG00000048828	0
rs4744242	9	93474429	*FAM120A*	ENSG00000048828	0
rs12377399	9	93481238	*FAM120A*	ENSG00000048828	0
rs34866682	9	93472223	*FAM120A*	ENSG00000048828	0
rs10891490	11	113014805	*NCAM1*	ENSG00000149294	0
rs7337807	13	98958327	*DOCK9*	ENSG00000088387	0
rs7338982	13	98958119	*DOCK9*	ENSG00000088387	0
rs7339076	13	98946874	*DOCK9*	ENSG00000088387	0
rs9513513	13	98943368	*DOCK9*	ENSG00000088387	0
rs9513514	13	98943672	*DOCK9*	ENSG00000088387	0
rs9513517	13	98952482	*DOCK9*	ENSG00000088387	0
rs9513519	13	98957892	*DOCK9*	ENSG00000088387	0

rs9517497	13	98942699	*DOCK9*	ENSG00000088387	0
rs9517508	13	98953938	*DOCK9*	ENSG00000088387	0
rs9517509	13	98955263	*DOCK9*	ENSG00000088387	0
rs4937872	11	112956992	*NCAM1*	ENSG00000149294	4282
rs2186710	11	112956325	*NCAM1*	ENSG00000149294	4949
rs12379660	9	93420205	*FAM120AOS*	ENSG00000188938	11235
rs10992729	9	93418793	*FAM120AOS*	ENSG00000188938	12647
rs12549729	8	8666402	*CLDN23*	ENSG00000253958	35534
rs622871	6	31910718	*C2*	ENSG00000166278	0
rs1052486	6	31642909	*BAG6*	ENSG00000204463	0
rs1077393	6	31642752	*BAG6*	ENSG00000204463	0
rs2077491	6	31638599	*BAG6*	ENSG00000204463	0
rs2077492	6	31638615	*BAG6*	ENSG00000204463	0
rs4569672	3	85007489	*CADM2*	ENSG00000175161	0
rs1248858	3	84978677	*CADM2*	ENSG00000175161	0
rs1248859	3	84967947	*CADM2*	ENSG00000175161	0
rs1694977	3	84987601	*CADM2*	ENSG00000175161	0
rs1872556	3	85016078	*CADM2*	ENSG00000175161	0
rs2035561	3	85006917	*CADM2*	ENSG00000175161	0
rs2035562	3	85007370	*CADM2*	ENSG00000175161	0
rs2326123	3	85016963	*CADM2*	ENSG00000175161	0
rs13081745	3	85014832	*CADM2*	ENSG00000175161	0
rs10156602	9	93583046	*PHF2*	ENSG00000197724	0
rs7105462	11	113041326	*NCAM1*	ENSG00000149294	0
rs7106434	11	112989857	*NCAM1*	ENSG00000149294	0
rs7947502	11	113038674	*NCAM1*	ENSG00000149294	0
rs2298527	11	112981239	*NCAM1*	ENSG00000149294	0
rs1248861	3	84958897	*CADM2*	ENSG00000175161	91
rs653481	3	84956534	*CADM2*	ENSG00000175161	2454
rs1248825	3	84944260	*CADM2*	ENSG00000175161	14728
rs426444	3	84943726	*CADM2*	ENSG00000175161	15262
rs382210	3	84916867	*CADM2*	ENSG00000175161	42121
rs2813573	13	53369198	*OLFM4*	ENSG00000102837	317140

The nearest gene was defined based on the shortest physical distance between the SNP and the transcription start or end site of protein-coding genes. Distances are shown in base pairs (bp).

Table S11. Bayesian colocalization results for IBS and blood pressure-related traits across candidate loci.

Gene	BP trait	Chr	Start	End	No. SNPs	PP.H0	PP.H1	PP.H2	PP.H3	PP.H4	Minimum P for IBS	Minimum P for BP trait	Top IBS SNP	Top BP SNP
CADM2	SBP	3	84008132	87123579	8632	0.000	0.000	0.001	0.985	0.015	1.20×10^{-9}	3.15×10^{-18}	rs1248825	rs12494446
	DBP	3	84008132	87123579	8643	0.000	0.000	0.001	0.980	0.019	1.20×10^{-9}	9.71×10^{-10}	rs1248825	rs2029134
	PP	3	84008132	87123579	8631	0.000	0.000	0.001	0.994	0.005	1.20×10^{-9}	9.24×10^{-16}	rs1248825	rs6803322
DOCK9	SBP	13	98445741	100738879	7178	0.003	0.788	0.001	0.207	0.001	3.09×10^{-8}	3.11×10^{-6}	rs9513519	rs72652239
	DBP	13	98445741	100738879	7184	0.004	0.935	0.000	0.059	0.002	3.09×10^{-8}	1.03×10^{-4}	rs9513519	rs7318325
	PP	13	98445741	100738879	7180	0.000	0.032	0.004	0.964	0.000	3.09×10^{-8}	2.32×10^{-8}	rs9513519	rs9554446
FAM120A	SBP	9	95214004	97328397	6169	0.000	0.000	0.001	0.999	0.000	4.36×10^{-9}	2.21×10^{-11}	rs10156602	rs7037075
	DBP	9	95214004	97328397	6170	0.000	0.339	0.000	0.659	0.001	4.36×10^{-9}	3.05×10^{-7}	rs10156602	rs1317939
	PP	9	95214004	97328397	6167	0.000	0.000	0.001	0.998	0.001	4.36×10^{-9}	7.64×10^{-12}	rs10156602	rs10761161
FAM120AOS	SBP	9	95208776	97215874	5768	0.000	0.000	0.001	0.999	0.000	4.36×10^{-9}	7.70×10^{-12}	rs10156602	rs10992340
	DBP	9	95208776	97215874	5769	0.000	0.340	0.000	0.659	0.001	4.36×10^{-9}	3.05×10^{-7}	rs10156602	rs1317939
	PP	9	95208776	97215874	5766	0.000	0.000	0.001	0.999	0.001	4.36×10^{-9}	2.10×10^{-12}	rs10156602	rs10992340
NCAM1	SBP	11	111831997	114149158	5955	0.000	0.000	0.000	0.225	0.774	3.19×10^{-8}	2.75×10^{-9}	rs7106434	rs11214436
	DBP	11	111831997	114149158	5971	0.000	0.004	0.000	0.120	0.875	3.19×10^{-8}	1.27×10^{-7}	rs7106434	rs11214436
	PP	11	111831997	114149158	5955	0.000	0.053	0.001	0.742	0.203	3.19×10^{-8}	2.42×10^{-7}	rs7106434	rs10891499
PHF2	SBP	9	95338689	97441869	6302	0.000	0.006	0.001	0.987	0.006	4.36×10^{-9}	3.64×10^{-9}	rs10156602	rs4744239
	DBP	9	95338689	97441869	6305	0.000	0.344	0.000	0.655	0.001	4.36×10^{-9}	3.05×10^{-7}	rs10156602	rs1317939
	PP	9	95338689	97441869	6300	0.000	0.000	0.001	0.983	0.016	4.36×10^{-9}	1.34×10^{-10}	rs10156602	rs4744239
WNK2	SBP	9	94947198	97082854	6185	0.000	0.000	0.001	0.999	0.000	4.36×10^{-9}	2.25×10^{-12}	rs10156602	rs7045409
	DBP	9	94947198	97082854	6186	0.000	0.338	0.000	0.660	0.001	4.36×10^{-9}	3.05×10^{-7}	rs10156602	rs1317939
	PP	9	94947198	97082854	6183	0.000	0.000	0.001	0.999	0.000	4.36×10^{-9}	8.19×10^{-13}	rs10156602	rs7045409

PP.H0 indicates the posterior probability of no association with either trait; PP.H1, association with IBS only; PP.H2, association with the blood pressure-related trait only; PP.H3, association with both traits through distinct causal variants; and PP.H4, association with both traits through a shared causal variant.

Table S12. SuSiE-based multi-signal colocalization analysis at the *NCAM1* locus

Trait	IBS signal	BP signal	PP.H3	PP.H4
DBP	rs7106434	rs11214436	0.097	0.903
SBP	rs7106434	rs648116	0.998	<0.001
SBP	rs7106434	rs11214783	0.983	<0.001
SBP	rs7106434	rs11214436	0.110	0.890

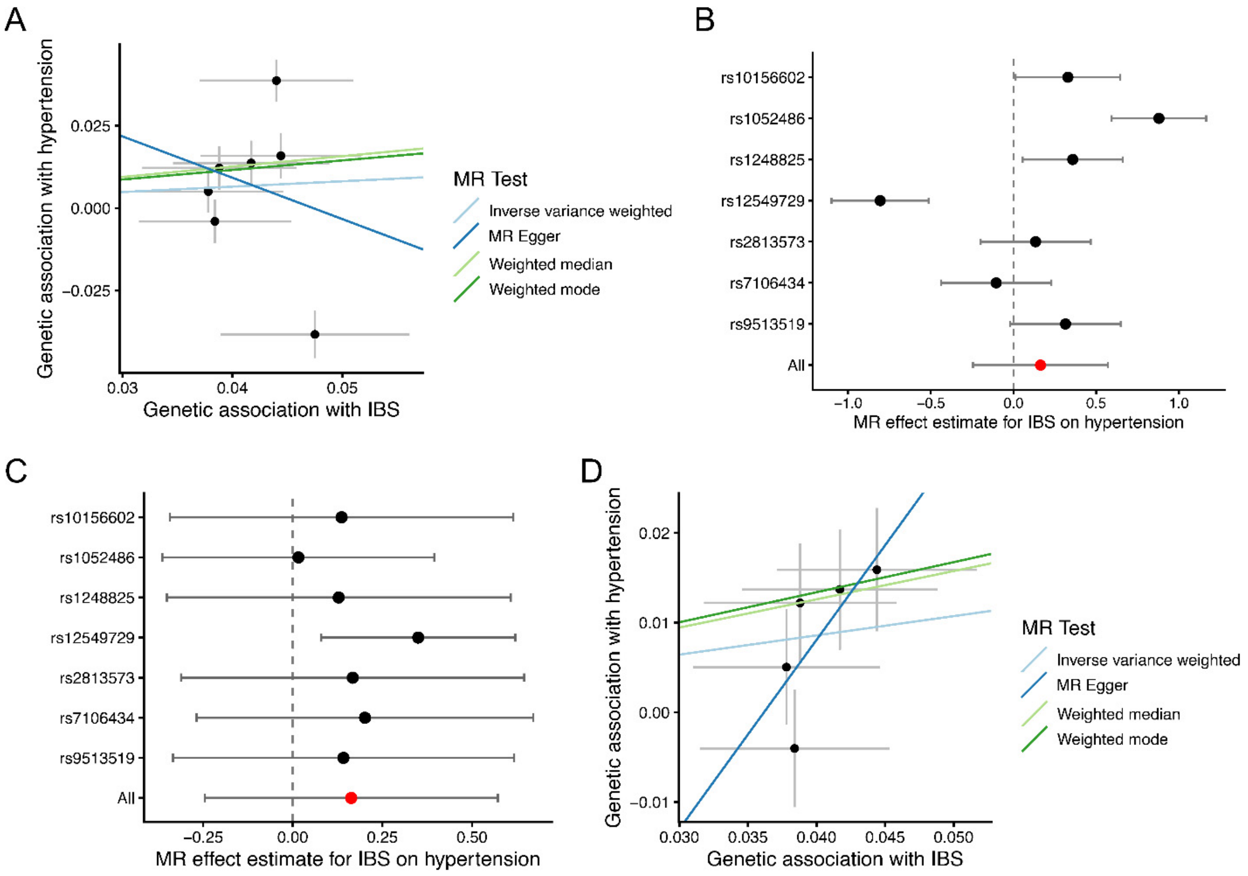

Figure S1. Mendelian randomization analyses and sensitivity analyses. (A) Scatter plot of SNP associations with IBS and hypertension, with estimates from IVW, weighted median, weighted mode, and MR-Egger methods using seven independent instruments. (B) Individual SNP estimates and the combined IVW estimate. (C) Leave-one-out analysis assessing the influence of individual instruments on the IVW estimate. (D) Scatter plot and MR estimates after exclusion of the two MR-PRESSO outliers, rs1052486 and rs12549729. Horizontal and vertical error bars in scatter plots indicate standard errors. IVW, inverse-variance weighted; MR, Mendelian randomization; SNP, single-nucleotide polymorphism.

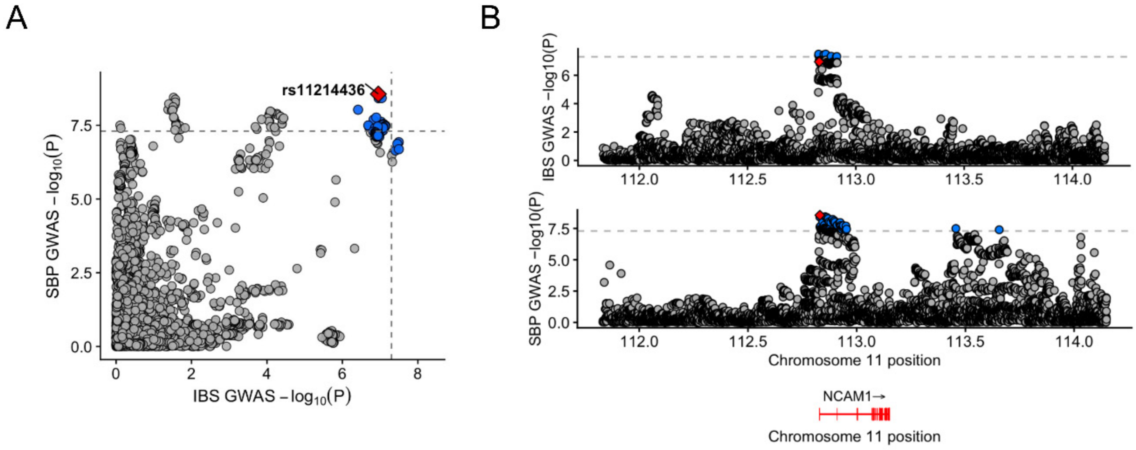

Figure S2. Colocalization plot of *NCAM1* and systolic blood pressure. (A) SNP-level comparison of IBS and SBP GWAS signals at the *NCAM1* locus. (B) Regional association plots of IBS and SBP GWAS signals at the *NCAM1* locus.

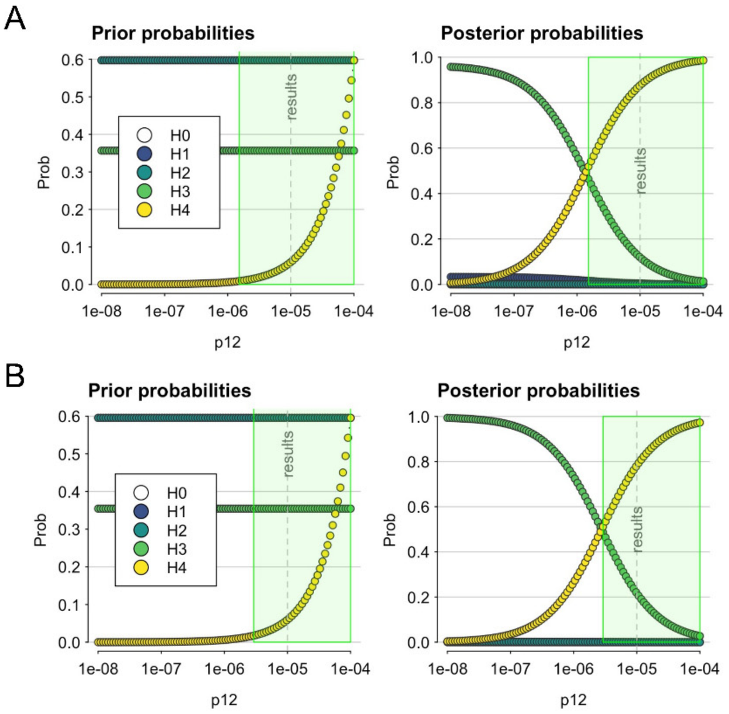

Figure S3. Prior sensitivity of colocalization at the *NCAM1* locus. Sensitivity analyses for colocalization between IBS and (A) diastolic blood pressure (DBP) and (B) systolic blood pressure (SBP). The prior probability that a variant is associated with both traits (p12) was varied from 10−8 to 10−4. Left panels show prior probabilities and right panels show posterior probabilities for hypotheses H0–H4: no association with either trait (H0), association with only one trait (H1/H2), associations driven by distinct causal variants (H3), or associations driven by a shared causal variant (H4). Vertical dashed lines indicate the default p12 of 10−5. Green shading indicates p12 values satisfying PP.H4 > 0.5 and PP.H4 > PP.H3. ——————————

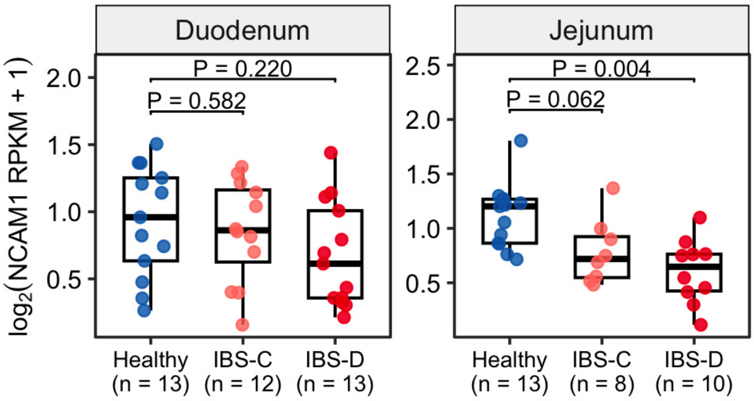

Figure S4. *NCAM1* expression across IBS subtypes in duodenal and jejunal mucosal samples. Box plots show the median and interquartile range, with individual samples overlaid. Sample sizes are indicated below each group. Pairwise *P* values were adjusted using the Benjamini–Hochberg method. IBS-C, constipation-predominant irritable bowel syndrome; IBS-D, diarrhea-predominant irritable bowel syndrome; RPKM, reads per kilobase of transcript per million mapped reads.
